# Evaluation of the risk of cardiovascular events with clarithromycin using both propensity score and self‐controlled study designs

**DOI:** 10.1111/bcp.12983

**Published:** 2016-06-08

**Authors:** Adrian A. Root, Angel Y. S. Wong, Yonas Ghebremichael‐Weldeselassie, Liam Smeeth, Krishnan Bhaskaran, Stephen J. W. Evans, Ruth Brauer, Ian Chi Kei Wong, Vidya Navaratnam, Ian Douglas

**Affiliations:** ^1^ Department of Epidemiology and Population HealthLondon School of Hygiene and Tropical MedicineLondonWC1E 7HTUK; ^2^ Centre for Safe Medication Practice and Research, Department of Pharmacology and PharmacyThe University of Hong KongHong Kong; ^3^ Department of Mathematics and Statistics, Faculty of Mathematics, Computing and TechnologyThe Open UniversityMilton KeynesBuckinghamshireMK7 6AAUK; ^4^ Research Department of Practice and Policy, School of PharmacyUniversity College LondonLondonWC1N 1AXUK

**Keywords:** adverse drug reaction, cardiovascular disease, clarithromycin, pharmacoepidemiology

## Abstract

**Aim:**

Some previous studies suggest a long term association between clarithromycin use and cardiovascular events. This study investigates this association for clarithromycin given as part of *Helicobacter pylori* treatment (HPT).

**Methods:**

Our source population was the Clinical Practice Research Datalink (CPRD), a UK primary care database. We conducted a self‐controlled case series (SCCS), a case–time–control study (CTC) and a propensity score adjusted cohort study comparing the rate of cardiovascular events in the 3 years after exposure to HPT containing clarithromycin with exposure to clarithromycin free HPT.

Outcomes were first incident diagnosis of myocardial infarction (MI), arrhythmia and stroke. For the cohort analysis we included secondary outcomes all cause and cardiovascular mortality.

**Results:**

Twenty‐eight thousand five hundred and fifty‐two patients were included in the cohort. The incidence rate ratio of first MI within 1 year of exposure to HPT containing clarithromycin was 1.07 (95% CI 0.85, 1.34, *P* = 0.58) and within 90 days was 1.43 (95% CI 0.99, 2.09 *P* = 0.057) in the SCCS analysis. CTC and cohort results were consistent with these findings.

**Conclusions:**

There was some evidence for a short term association for first MI but none for a long term association for any outcome.

## What is Already Known about this Subject

- Previous epidemiological studies suggest that clarithromycin is associated with an increased risk of cardiovascular events at least 1 year after exposure.
- A recent study in a Hong Kong population suggests that there is no long term risk, only a short term risk associated with currently taking the drug.

## What this Study Adds

- This study corroborates the findings of the Hong Kong study in a larger UK population that has been well validated for cardiovascular outcomes.
- Clarithromycin is not associated with a long term increased risk of cardiovascular events.
- There is some evidence for an increased short term risk.

## Introduction

Clarithromycin is a very commonly prescribed antibiotic in both primary and secondary care settings. As well as having specific indications, it is one of the most commonly prescribed alternatives for patients allergic to penicillin. The summary of product characteristics states that clarithromycin, along with other macrolides, can cause QT prolongation and thereby increase the short term risk of cardiac arrhythmias. However two recent papers have suggested an association between clarithromycin exposure and a broad range of subsequent cardiovascular events that extends for at least 1 year after taking the course of medication 1, 2. This is incompatible with temporary QT prolongation being the underlying mechanism and if this association were causal, it could have profound implications for clarithromycin prescribing.

It is possible that in previous studies people given clarithromycin were generally frailer than people given other antibiotics despite correction for measured confounders. This type of indication bias is common in observational studies of drug effects and can lead to findings of non‐causal associations. To avoid this, we have chosen to restrict our investigation to the association between clarithromycin given as part of *Helicobacter pylori* treatment (HPT) and subsequent cardiovascular events. The restriction to HPT regimes should reduce confounding by indication, as the choice of HPT regime is unlikely to be closely linked with a patient's underlying risk of cardiovascular outcomes. Furthermore, we employed three study designs with complementary strengths and weaknesses to guard further against conclusions based on potentially biased results. A causal association should show a consistent pattern across study designs whereas discordant findings may suggest important bias. Finally, we have completed this study protocol in a Hong Kong population cohort to ensure generalizability to different ethnicities and to guard against biases derived from a single health care database. 3.

## Methods

### Clinical Practice Research Datalink (CPRD)

The CPRD is a large UK primary care electronic healthcare records database widely validated for epidemiological research 4. A subset of the CPRD database has been linked to the Office of National Statistics (ONS) and Hospital Episodes Statistics (HES) databases which provide cause of death data and hospital discharge information, respectively. We used the full CPRD database for all outcomes except for mortality outcomes where we used this linked subset.

### Selection of participants

Patients were selected from the population registered at participating general practices that were up to research standard before January 2014. All patients exposed to HPT during the registration period were included. Patients who had either exposure or outcome recorded during their first year of registration in the database were excluded from the cohort since records entered close to registration could reflect historic data.

### Exposure

Exposure to HPT was determined by prescription for all three components of a triple therapy regime listed in the British National Formulary (BNF) on the same day. It was considered very unlikely to receive this particular combination of drugs for any other indication. We included patients who received courses of treatment lasting between 1 and 2 weeks duration. Patients who received a prescription for a HPT regime containing clarithromycin (CHPT) were the exposed group and for the cohort design, patients who received a prescription for a clarithromycin free HPT regime (NHPT) were the unexposed comparator group. The comparator group was chosen to minimize the risk of indication bias as both regimes have the same indication. All regimes were taken from the BNF and are listed in Appendix S1. There were insufficient patients with a specific Read code for *H. pylori* infection to use these in our exposure definition. However, we conducted a sensitivity analysis using the subset of patients who also had a Read code for *H. pylori* to validate our approach.

### Outcomes

First recorded incident diagnosis of myocardial infarction (MI), arrhythmia and stroke were analyzed as separate outcome measures for all three study designs. These outcomes were selected as they were components of the composite outcomes reported by Schembri *et al.* 2. All subsequent diagnoses of the same event type were excluded to reduce the possibility of a repeated entry of the same event. The validity of recording MI in the CPRD has previously been confirmed by Herrett *et al.* 5. However, they described a small delay between MI events coded in CPRD compared with the same events coded in HES. It is possible that this delay might either reduce the power of our analysis or result in a delayed association being found. This would particularly affect the self‐controlled case series method and we conducted a sensitivity analysis using this method in a subset of CPRD patients who have linked HES records using HES MI dates.

All cause mortality and cardiovascular mortality were included for the cohort design only since the self‐controlled methods would be biased for this outcome. Cardiovascular mortality was obtained from linked ONS data, which was available from 1 January 1998 to 10 January 2012 for a subset of CPRD.

### Propensity adjusted cohort study

Patients entered this study from the day they first received a prescription for any form of HPT. They were followed up for 3 years. For all patients in the cohort follow‐up was censored at the first date of any of the following: leaving the CPRD, death, last data collection from the general practitioner (GP) or at the next prescription for clarithromycin either alone or as part of HPT.

A Poisson regression model was used to measure the rate ratio of outcome occurrence for those exposed to CHPT compared with NHPT. To control for confounding, a propensity score was developed as detailed in Appendix S2. This was included as a covariate in the final outcome model. For the variables smoking status, alcohol status and Body Mass Index (BMI), there were some missing data and this was analyzed by creating an unknown category. A sensitivity analysis using just complete records was carried out. The distribution of propensity scores for both groups was examined (Appendix S3). All patients whose scores fell outside of the overlapping region of both distributions were removed from the outcome model. In addition the top and bottom 5% of each distribution was removed from the outcome model. These adjustments were made because people treated contrary to extreme scores may have important unmeasured characteristics that could bias effect estimates 6. We conducted a sensitivity analysis without trimming the 5% tails of each distribution to investigate the effect of this analysis decision.

A secondary analysis was performed where the study period was stratified by time since exposure into time windows. These strata were days 1–90, days 91–365, days 366–730 (years 1–2) and days 731–1095 (years 2–3) post‐exposure. This analysis was designed to model any change in risk over time. There was insufficient power to look at shorter initial risk periods.

### Self‐controlled case series study (SCCS)

This study design is derived from rate modelling using a Poisson distribution and is analogous to cohort methodology. It relies on within person comparisons in a population with both the cardiovascular event outcome and exposure to CHPT 7, 8 Incidence rate ratios are derived, comparing the rate of cardiovascular events during predefined risk periods following exposure to CHPT with that during all other observed periods. In this case the risk period was defined as the first year following exposure in this analysis. A major advantage of this design is that it removes the potential confounding effect of both recorded and unrecorded time invariant characteristics between people. Age, which varies over time, was adjusted for in the analysis (age bands are detailed in Appendix S4). The method relies on several assumptions. These assumptions and our approach to handling them are detailed in Appendix S5.

For this analysis, follow‐up was from 1 year following registration with the database until the patient died, moved to a different general practice or the last data collection by the practice before January 2014. As with the cohort design, a secondary analysis was undertaken where several risk windows post‐exposure were compared with the baseline rate: days 1–30, days 31–90, days 91–365, days 366–730 (years 1–2) and days 731–1095 (years 2–3) post‐exposure.

Finally we employed a non‐parametric SCCS design using cubic splines that does not require a pre‐specified risk period to model the association between CHPT and first MI. This method allows better visualization of the profile of relative risk over time 9.

### Case–time–control study (CTC)

This design, described by Suissa 10, is a variation of the case–crossover study that controls for possible changes in exposure trends over time. The comparison is between a case period and a reference period within the same patient and the control patients are used to remove any bias from underlying prescription trends. Controls were matched on gender, age to the nearest year, general practice and registration period.

A conditional logistic regression model including the interaction between the case/control indicator and the time period indicator variables was performed. In this model, the effect of the exposure is given by the interaction term whereas the effect of the time period in the absence of exposure is given by the time period term.

### Data analysis and power considerations

All analyses were conducted using Stata software, version 13 (StataCorp, College Station, TX, USA). Prior to undertaking the analyses we estimated that we would have over 99% power to detect a relative risk of 1.5 and 80% power to detect a relative risk of 1.3 for the cohort analysis assuming the 1 year risk of MI is 4/1000 in adults (Coronary Heart Disease Statistics 2010, BHF).

### Ethics

Ethical approval was granted by the London School of Hygiene and Tropical Medicine Ethics Committee (PR/203/203) and scientific approval was granted by the Independent Scientific Advisory Committee of the Medicines and Healthcare Products Regulatory Agency (ISAC Reference 14_066R).

## Results

### Cohort study

**Figure 1:**
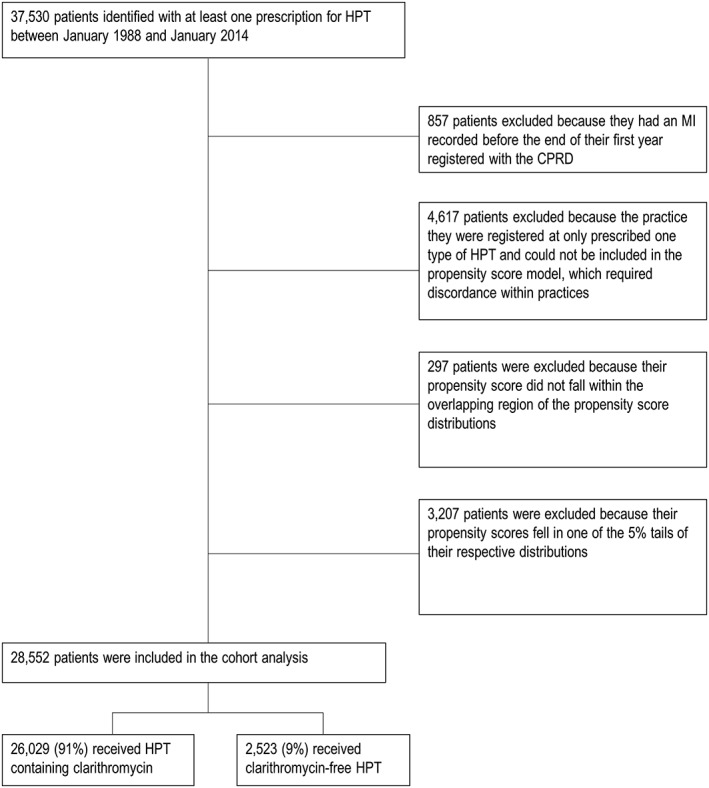
Flow chart for the propensity score adjusted cohort analysis of first MI

We identified 37 530 patients in the database with at least one prescription of HPT. Figure 1 is a flow diagram of patients excluded from the cohort. Twenty‐eight thousand five hundred and fifty‐two patients were included in the analysis. Of these 26 029 (91%) received CHPT and 2523 (9%) received NHPT. For both groups the mean age at exposure was 53 years and the median follow‐up was 3 years. For the CHPT group the mean age at first MI was 67 years compared with 69 years for the NHPT group. Table 1 shows the baseline characteristics for these groups (Appendix S6 shows the baseline characteristics for the cohort before the exclusions listed above). There were no large differences between the two groups on any characteristic.

**Table 1:** Baseline characteristics for patients included in the cohort study between 1991 and 2013 with a median follow‐up of 3 years

**Characteristic**	**Clarithromycin containing HPT regime**	**Clarithromycin‐free HPT regime**
	***n* (%)**	***n* (%)**
**Gender**		
**Male**	12386 (47.6%)	1196 (47.4%)
**Female**	13643 (52.4%)	1327 (52.6%)
**Age**		
**0–40 years**	6114 (23.5%)	592 (23.5%)
**40–50 years**	5365 (20.6%)	518 (20.5%)
**50–60 years**	5343 (20.5%)	529 (21.0%)
**60–70 years**	4976 (19.1%)	478 (19.0%)
**70–80 years**	3135 (12.0%)	301 (11.9%)
**>80 years**	1096 (4.2%)	105 (4.2%)
**Smoking status**		
**non‐smoker**	10392 (39.9%)	1052 (41.7%)
**current smoker**	7409 (28.5%)	632 (25.1%)
**ex‐smoker**	7884 (30.3%)	812 (32.2%)
**unknown**	344 (1.3%)	27 (1.1%)
**Alcohol status**		
**non‐drinker**	4330 (16.6%)	442 (17.5%)
**ex‐drinker**	1133 (4.4%)	120 (4.8%)
**current drinker (unknown quantity)**	102 (0.4%)	11 (0.4%)
**<2 u day^–1^**	4387 (16.9%)	447 (17.7%)
**3‐6 u day^–1^**	10900 (41.9%)	1016 (40.3%)
**>6 u day^–1^**	2588 (9.9%)	234 (9.3%)
**unknown**	2589 (10.0%)	253 (10.0%)
**Body mass index (kg m^−2^)**		
**normal (18.5–25)**	9642 (37.0%)	941 (37.3%)
**overweight (25–30)**	8884 (34.1%)	856 (33.9%)
**obese I (30–35)**	3585 (13.8%)	367 (14.6%)
**obese II (35–40)**	1109 (4.3%)	115 (4.6%)
**obese III (>40)**	472 (1.8%)	44 (1.7%)
**unknown**	2337 (9.0%)	1200 (7.9%)
**Consulted GP in year before exposure**	25948 (99.7%)	2514 (99.6%)
**History of cardiovascular disease**	4226 (16.2%)	366 (14.5%)
**History of heart failure**	755 (2.9%)	59 (2.3%)
**History of arrhythmia**	1434 (5.5%)	127 (5.0%)
**History of hypertension**	8240 (31.7%)	783 (31.0%)
**History of COPD**	1682 (6.5%)	167 (6.6%)
**History of asthma**	3615 (13.9%)	350 (13.9%)
**History of hyperlipidaemia**	4605 (17.7%)	416 (16.5%)
**History of diabetes mellitus**	3734 (14.4%)	342 (13.6%)
**History of cancer**	4884 (18.8%)	461 (18.3%)
**History of NSAID use**	1942 (7.5%)	167 (6.6%)
**History of oral corticosteroid use**	383 (1.5%)	36 (1.4%)
**History of antipsychotic use**	854 (3.3%)	81 (3.2%)
**History of antidepressant use**	3101 (11.9%)	294 (11.7%)
**History of lipid lowering drug use**	2948 (11.3%)	336 (13.3%)
**History of anticoagulant use**	226 (0.9%)	32 (1.3%)
**History of antiplatelet use**	2156 (8.3%)	242 (9.6%)
**History of nitrate use**	649 (2.5%)	56 (2.2%)
**History of digoxin use**	146 (0.6%)	15 (0.6%)
**History of antiarrhythmic drug use**	59 (0.2%)	6 (0.2%)
**History of β‐adrenoceptor blocker use**	2192 (8.4%)	230 (9.1%)
**History of thiazide diuretic use**	1892 (7.3%)	181 (7.2%)
**History of calcium channel blocker use**	2072 (8.0%)	216 (8.6%)
**History of ACEI/ARB use**	2843 (10.9%)	316 (12.5%)
**History of loop diuretic use**	718 (2.8%)	62 (2.5%)
**Total**	26029	2523

ACEI angiotensinc enzyme inhibitor, ARB angiotensin receptor blocker, COPD chronic obstructive pulmonary disease, HPT *Helicobacter pylori* treatment, NSAID non‐steroidal anti‐inflammatory drug

Table 2 shows the results of Poisson regression analysis. For first MI, the rate ratio for CHPT compared with NHPT exposure was 0.75 (95% CI 0.45, 1.24, *P* = 0.26) after propensity score adjustment. There was no association.

**Table 2:** Results of the propensity score adjusted cohort analysis using Poisson regression

	**Patients (*n*)**	**Patient‐years**	**Events (*n*)**	**Crude IRR (95% CI)**			**PS Adjusted IRR (95% CI)**		
**First MI**									
**CHPT**	26 029	62118.98	174	0.89	(0.54, 1.44)	*P* = 0.62	0.75	(0.45, 1.24)	*P* = 0.26
**NHPT**	2523	5688.98	18	1.00					
**First stroke**									
**CHPT**	26 686	63847.36	68	0.38	(0.22, 0.66)	*P* = 0.001	0.47	(0.26, 0.84)	*P* = 0.01
**NHPT**	2540	5746.98	16	1.00					
**First arrhythmia**									
**CHPT**	26 586	63581.67	95	0.43	(0.26, 0.69)	*P* = 0.001	0.37	(0.22, 0.63)	*P* = 0.001
**NHPT**	2527	5702.77	20	1.00					
**All cause mortality**									
**CHPT**	26 827	64235.69	2621	1.09	(0.95, 1.25)	*P* = 0.22	0.97	(0.84, 1.12)	*P* = 0.66
**NHPT**	2582	5851.81	219	1.00					
**Cardiovascular mortality**									
**CHPT**	11 616	27729.71	416	1.05	(0.73, 1.50)	*P* = 0.80	0.93	(0.64, 1.34)	*P* = 0.69
**NHPT**	1058	2234.28	32	1.00			1.00		

CHPT *Helicobacter pylori* treatments containing clarithromycin, CI confidence interval, HPT *Helicobacter pylori* treatment, IRR incidence rate ratio, MI myocardial infarction, NHPT clarithromycin free *Helicobacter pylori* treatment

For first arrhythmia the adjusted rate ratio was 0.37 (95% CI 0.22, 0.63, *P* = 0.001).

For first stroke the adjusted rate ratio was 0.47 (95% CI 0.26, 0.84, *P* = 0.01). There was good evidence that CHPT was associated with a reduced incidence of both first arrhythmia and first stroke. For all cause mortality and cardiovascular mortality there was no evidence of an association in any of the analyses (see Table 2).

Appendix S7 shows the results for all outcomes stratified by time. There was some evidence for a protective association for first arrhythmia between years 1 and 2 post‐exposure.

### Self‐controlled case series study (SCCS)

**Figure 2:**
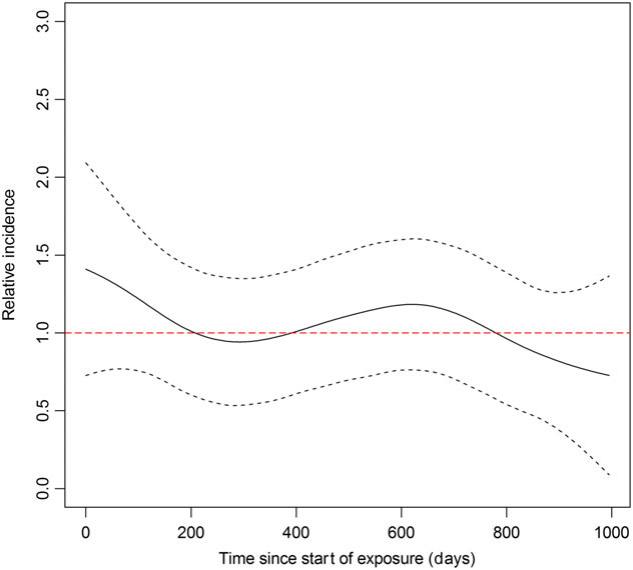
Non‐parametric self‐controlled case series analysis of the relative incidence of first myocardial infarction after exposure to clarithromycin containing *Helicobacter pylori* therapy. The black dashed lines represent upper and lower 95% confidence intervals surrounding the relative incidence estimate. The red dashed line represents the null value

Nine hundred and sixty‐two patients were both exposed to CHPT and had a first MI within the registration period in CPRD. They had a mean follow‐up time of 14 years. The age adjusted rate ratio for incident first MI in the year after exposure to CHPT compared with the rest of follow‐up was 1.07 (95% CI 0.85, 1.34, *P* = 0.58). There was no association between CHPT and first MI in the first year after taking it. In the secondary analysis comparing multiple risk windows over the 3 years following exposure to baseline there was some evidence of an increased risk at year 1 to 2 post‐exposure with a rate ratio of 1.27 (95% CI 1.01, 1.61, *P* = 0.04). These results are shown in Table 3. A non‐parametric SCCS analysis showed no association between exposure to CHPT and first MI and this is shown in Figure 2. As the risk windows 1–30 days post‐exposure and 31–90 days post‐exposure contained very few events these were combined *post hoc* to improve power. The incidence rate ratio for days 1–90 post‐exposure was 1.43 (95% CI 0.99, 2.09, *P* = 0.057), suggesting a possible association between exposure to CHPT and subsequent MI within 90 days.

**Table 3:** Results of the self‐controlled case series analysis for the outcomes of first MI and first arrhythmia

	**Patients (*n*)**	**Patient‐years**	**Events (*n*)**	**Age adjusted IRR (95% CI)**		
**Primary outcome: First MI (median follow‐up 14.0 years)**						
**Single risk window**						
**Baseline**	962	12 718	876	1		
**1 year post‐exposure**	961	932.5	84	1.07	(0.85, 1.34)	*P* = 0.58
**Multiple risk window**						
**Baseline**	962	11 104	731	1		
**days 1–30 post‐exposure**	961	81.08	9	1.32	(0.6, 2.55)	*P* = 0.41
**days 31–90 post‐exposure**	954	159.42	20	1.50	(0.96, 2.35)	*P* = 0.08
**days 91–365 post‐exposure**	941	694.45	55	0.97	(0.74, 1.29)	*P* = 0.84
**years 1–2 post‐exposure**	886	843.08	85	1.27	(1.01, 1.61)	*P* = 0.04
**years 2–3 post‐exposure**	800	768.74	60	1.01	(0.77, 1.33)	*P* = 0.92
**Secondary outcome: first arrhythmia (median follow‐up 15.0 years)**						
**Single risk window**						
**Baseline**	552	7727.83	498	1		
**1 year post‐exposure**	552	542.11	50	1.24	(0.92, 1.68)	*P* = 0.16
**Multiple risk window**						
**Baseline**	552	6761.63	432	1		
**days 1–30 post‐exposure**	552	46.57	5	1.42	(0.58, 3.44)	*P* = 0.44
**days 31–90 post‐exposure**	548	91.83	14	2.04	(1.19, 3.51)	*P* = 0.01
**days 91–365 post‐exposure**	543	405.14	31	0.99	(0.68, 1.45)	*P* = 0.97
**years 1–2 post‐exposure**	514	500.77	35	0.89	(0.63, 1.27)	*P* = 0.53
**years 2–3 post‐exposure**	473	464	31	0.83	(0.57, 1.2)	*P* = 0.33

All IRRs are age adjusted and derived from conditional Poisson regression. MI myocardial infarction, CI confidence interval, IRR incidence rate ratio

Five hundred and fifty‐two patients were both exposed to CHPT and had a first arrhythmia within the registration period in CPRD. They had a mean follow‐up time of 15 years. The age adjusted rate ratio for incident first arrhythmia in the year after exposure to CHPT compared with the rest of follow‐up was 1.24 (95% CI 0.92, 1.68, *P* = 0.16). There was no association between CHPT and first arrhythmia in the first year after taking it. In the secondary analysis comparing multiple risk windows over the 3 years following exposure to baseline there was evidence of an increased risk from days 30–90 post‐exposure with a rate ratio of 2.04 (95% CI 1.19, 3.51, *P* = 0.01). There was no evidence of an increased risk during other time windows examined.

Appendix S8 shows the SCCS analysis for first stroke. There was no evidence of an association between CHPT and increased risk of stroke.

### Case–time–control study (CTC)

Eighty‐two thousand seven hundred and eight patients had a first MI during the registration period. These were matched to controls aiming for 4 : 1 matching. Seven thousand seven hundred and ninety‐seven patients did not have a suitable match and were excluded from the analysis. One hundred and forty‐two patients were excluded because they did not have 3 years of follow‐up before the first MI. The remaining 74 769 cases were matched to 258 696 controls.

The odds ratio for exposure to CHPT in the year before first MI compared with the reference period between 1 and 2 years before MI was 0.86 (95% CI 0.59, 1.26, *P* = 0.44). There was no association between exposure to CHPT and first MI within a year. We carried out a *post hoc* analysis comparing the current period 0–90 days before first MI with a reference period 91–180 days before first MI to mirror the *post hoc* SCCS analysis described above. The odds ratio comparing these periods was 1.32 (95% CI 0.62, 2.80, *P* = 0.74).

There was no association between exposure to CHPT and either first arrhythmia or first stroke. Table 4 shows the results of the CTC analysis for all outcomes.

**Table 4:** Results of case‐time‐control analysis for all outcomes

	**Patients (*n*)**	**Patient‐years**	**Events (*n*)**	**OR (95% CI)**		
**First MI**						
**Exposure effect**	33 465	737 176	74 769	0.86	(0.59, 1.26)	*P* = 0.44
**Period effect**				1.08	(0.90, 1.31)	*P* = 0.41
**Case‐crossover equivalent**				0.93	(0.67, 1.29)	*P* = 0.68
**First stroke**						
**Exposure effect**	53 430	108 812	11 025	1.17	(0.58, 2.36)	*P* = 0.67
**Period effect**				1.10	(0.67, 1.82)	*P* = 0.70
**Case‐crossover equivalent**				1.29	(0.78, 2.11)	*P* = 0.32
**First arrhythmia**						
**Exposure effect**	87 256	179 566	18 137	1.46	(0.79, 2.70)	*P* = 0.23
**Period effect**				0.96	(0.64, 1.43)	*P* = 0.84
**Case‐crossover equivalent**				1.40	(0.88, 2.24)	*P* = 0.16

Conditional logistic regression analysis. Exposure effect OR for the effect of exposure after adjusting for differences in prescription patterns between the two periods. Period effect OR for period indicator variable: this represents the effect of the difference in prescription rates between the two periods that is not due to exposure effects. Case‐crossover equivalent crude OR for exposure period compared with reference period: this represents the total effect comparing periods before adjusting for differences between periods due to underlying prescription patterns i.e. a simple case‐crossover analysis. OR odds ratio, MI myocardial infarction, CI confidence interval.

### Sensitivity analyses

Three hundred and fifty‐nine CPRD patients with linked HES records were exposed to clarithromycin containing HPT and had a first MI event within follow‐up. An SCCS analysis on this cohort using event dates recorded in HES showed an age adjusted rate ratio for incident first MI in the 30 days after exposure to CHPT compared with the rest of follow‐up of 3.77 (95% CI 1.85, 7.68, *P* < 0.001). There was no association with any other time periods. The results are shown in Appendix S9.

This sensitivity analysis was not conducted for the first arrhythmia outcome because the databases were often discordant with respect to the first arrhythmia event. In particular, many first arrhythmias were coded in CPRD but not in HES which probably reflects the fact that many of these cases did not require inpatient admission. These discordant events might possibly reflect historical events coded more recently.

None of the other sensitivity analyses conducted conflicted with our main analyses. The results are not shown.

## Discussion

This study found no evidence that clarithromycin in the context of HPT was associated with the first MI within 1 year of exposure. There was, however, some evidence of a short lived increased risk of first MI and first arrhythmia within 90 days of exposure in the SCCS. The statistical evidence for this result was weak and it should be treated with caution. In particular, arrhythmia events were discordant when compared with hospital data and although this is likely to represent milder arrhythmias that did not require hospital admission the possibility remains that there was increased case finding by clinicians aware of recent clarithromycin use and the potential association with arrhythmia.

Despite these caveats, it is consistent with the summary of product characteristics document for clarithromycin, which lists prolonged QT interval as a recognized side effect. Prolonged QT interval is a cause of arrhythmia and arrhythmia in turn can precipitate MI.

The sensitivity analysis of patients with linked HES records with more accurate HES outcome dates revealed a strong short term effect within 30 days of exposure. This suggests that there is some temporal lag in CPRD event recording and the true risk period might be much shorter.

The cohort analysis suggested a protective effect of CHPT on the incidence of first stroke and first arrhythmia. However this finding would not be predicted by the known pharmacology of clarithromycin. Moreover it was not confirmed by the SCCS or CTC analyses and should be viewed with caution. Clinicians will be aware of the association between clarithromycin and prolonged QT interval. It is possible that patients at high risk of ventricular arrhythmia, for example with a relevant family history, would be prescribed NHPT preferentially and this would manifest as a protective effect in a cohort analysis comparing patients prescribed CHPT with patients prescribed clarithromycin free HPT. This may not be captured by the propensity score for two reasons. Firstly, there is likely to be significant under‐reporting of risk factors for arrhythmia such as family history of ventricular arrhythmia in the CPRD. There were less than five patients in the cohort with a code for this. Secondly, the propensity score adjusted for history of any arrhythmia. This includes all subtypes and is dominated by atrial fibrillation. This is an imperfect covariate. However, as there were only 10 patients in the cohort with codes for ventricular arrhythmia or long QT syndrome, including a more specific covariate was not feasible in this study. Since arrhythmia is a cause of stroke this could also be a cause of the protective effect seen for stroke also. Comparing the discordant results of the cohort and the self‐controlled methods we feel that it is more plausible that the cohort suffers from residual uncontrolled confounding than the alternative explanation that clarithromycin is protective for arrhythmia and stroke and that the self‐controlled designs were biased towards the null.

### Comparison between study designs

All three methods were consistent in not finding any long term harmful association between CHPT and any of the study outcomes.

The SCCS analysis showed some evidence of short term risk of MI and arrhythmia that was not demonstrated in the cohort analysis. However, the cohort analysis lacked power as evidenced by very wide confidence intervals which were unable to rule out potentially large effects. A *post hoc* CTC analysis of the short term risk period for MI suggested an effect estimate consistent with the SCCS but with confidence intervals crossing unity.

### Strengths and weaknesses

The strengths of this study are that it draws from a large representative primary care population and therefore is generalizable to the UK population. The exposure is restricted to an indication which is unlikely to be biased by acute infection and it employs several analytic methods with different susceptibilities to bias to answer the same question which reduces the risk that congruent findings across methods are due to bias.

A weakness of the cohort analysis was that the NHPT group was much smaller than the CHPT group. This compromised the power of this analysis. NHPT regimes all contain metronidazole. It is likely that these are less often prescribed because metronidazole is more likely to cause gastrointestinal side effects such as nausea and vomiting. Additionally, the BNF recommends these regimes as second line and so they are likely to be prescribed only for patients with allergy to HPT regimes containing clarithromycin. We do not know of any reason why this prescribing behaviour would result in differences in baseline cardiovascular risk between groups and the baseline characteristics measures were similar (Table 1). The consistency with the two self‐controlled analyses suggests that any bias from this is unlikely to have significantly affected the analysis.

The CTC analysis compared the first MI in the year following exposure to a baseline period between 1 and 2 years following exposure. This would be sensitive to a risk within the year following exposure but would underestimate a longer term risk as this would make the exposure period more similar to the baseline period.

The SCCS analysis can be biased if the outcome event causes significant censoring of subsequent exposures. This can occur with events that are associated with subsequent death. Although there is an increased mortality following first MI this represented a small proportion of the cohort (less than 10% died in the year after first MI). Previous studies have shown that the increased mortality following first MI is not sufficient to cause significant bias 11, 12. We performed a sensitivity analysis excluding patients who died in the first 30 days following first MI and found no difference in the study estimates.

This study was restricted to clarithromycin given as part of HPT. While this restriction was employed to reduce confounding by acute infection, the results are only strictly applicable to this particular indication. However, there is no good reason to suppose that adverse effects of taking clarithromycin would differ by indication.

For the outcome of first arrhythmia, there is already evidence that clarithromycin prolongs the QT interval and this would be expected to cause certain arrhythmia subtypes. In this study we do not have sufficiently detailed data on arrhythmia subtype to confirm whether the short term association we reported was entirely due to this potentially causal mechanism. If this were the only underlying causal mechanism, our broad outcome definition of all first arrhythmias would be expected to underestimate the strength of this causal association with specific arrhythmia subtypes such as torsades de pointes.

The outcomes measured in this study were first occurrence of the respective cardiovascular event. Therefore, the findings of this study are only strictly applicable to patients with no history of that particular cardiovascular event. However, other well established cardiovascular risk factors, such as hypertension and smoking, carry the same relative risk regardless of a patient's past medical history. Therefore, there are no grounds to suspect a different effect from exposure to clarithromycin for patients with a cardiovascular event history compared with those who have no such history.

### Comparison with previous studies

Jespersen conducted an RCT investigating the possible benefit of clarithromycin in secondary prevention of MI 1. None of the primary or secondary outcomes of the study showed any effect. However they reported an increased risk of both cardiovascular mortality and a tertiary composite outcome (including cardiovascular mortality, MI, stroke, unstable angina and peripheral vascular disease). This association could therefore be vulnerable to multiple testing.

Schembri *et al*. reported two cohorts comparing clarithromycin with other antibiotics to treat pneumonia and infective chronic obstructive pulmonary disease exacerbations, respectively 2. It is possible that this study was susceptible to indication bias where frailer patients could have been preferentially given clarithromycin over comparator antibiotics such as amoxicillin and this frailty might not have been adequately captured by the measured covariates.

Svanstrom *et al.* performed a propensity score adjusted cohort analyses comparing the risk of cardiac death after exposure to clarithromycin with exposure to penicillin V 13. They found an increased risk of cardiac death during current use (adjusted rate ratio 1.76, 95% CI 1.08, 2.85) that did not persist in the 30 days following the end of treatment. They repeated the analysis substituting roxithromycin for clarithromycin and did not find any association (adjusted rate ratio 1.04, 95% CI 0.72, 1.51). They concluded that clarithromycin was associated with an acute cardiac risk that did not persist after treatment was stopped. In this study there were clear baseline differences between the clarithromycin group and the penicillin V control group, the latter being younger, on less medication and having less respiratory illness. Therefore, the acute risk could have been related to these baseline differences.

Finally, we looked at this association in a Hong Kong population employing a similar protocol 3. Due to the smaller size of the Hong Kong database the cohort method could not be applied to *H. pylori* treatment only. Instead, the self‐controlled methods were applied to a *H. pylori* treatment cohort and a propensity score controlled cohort method was used to compare clarithromycin with amoxicillin for any indication. This study showed increased risk of MI during current use of clarithromycin in all study designs but no long term risk after finishing the course. As with the study by Schembri *et al.* 2 discussed above, the cohort method is susceptible to indication bias. However, the congruence with self‐controlled methods makes this less likely here.

Our cohort was slightly younger than the first two papers (mean 53 years compared with 65 and 72 years, respectively) and comparable with the latter two. The spread of ages in our study was appreciably wider (SD 16 years compared with 10.3 years in Jespersen 1 and 9.6 years in Svanstrom *et al.* 13). This suggests that our study encompasses a broader cross‐section of the population than the previous studies.

The study we report does not confirm the long term risk of clarithromycin suggested by Jespersen 1 and Schembri *et al.* 2. However, we cannot rule out a short term increased risk of MI and arrhythmia, which is consistent with Svanstrom *et al.* 13 and Wong *et al.* 3.

### Clinical implications

Clarithromycin is widely used in UK primary care for a range of indications. The suggestion of a raised long term cardiovascular risk was therefore a major concern. This study does not support this long term association. The SCCS analysis was compatible with a short term risk of both MI and arrhythmia, as expected, given the known pharmacology of clarithromycin. The SCCS method cannot directly provide absolute estimates of risk. However, if the risk of MI in the cohort group taking NHPT is used as a baseline comparator, this analysis would be compatible with an absolute rate increase for MI of three events per 10 000 treatment courses (assuming a maximum risk period of 90 days per course). For first arrhythmia the absolute rate increase would be eight events per 10 000 treatment courses. At present, the Summary of Product Characteristics for clarithromycin advises caution when prescribing clarithromycin in patients with coronary heart disease and recommends not prescribing clarithromycin to patients with a history of ventricular arrhythmia.

In conclusion this study found no long term association between clarithromycin prescribed as part of HPT and cardiovascular events in a large UK primary care cohort. Our results are consistent with a short term increased risk of MI and arrhythmia within 90 days of exposure.

## Availability of data and materials

We are not able to share the data from this study under the terms of use of the CPRD. However, an application can be made directly to the independent scientific advisory committee to access this data for research purposes. All code lists are available on request to the corresponding author.

## Competing Interests

All authors have completed the Unified Competing Interest form at http://www.icmje.org/coi_disclosure.pdf (available on request from the corresponding author) and declare ID holds stock in and consults for GlaxoSmithKline. All other authors declare no financial relationships with any organizations that might have an interest in the submitted work in the previous 3 years and no other relationships or activities that could appear to have influenced the submitted work.

*We are grateful to Miss Fiona Justice for proof reading the final draft.*

## Contributors

AR, AW, LS and ID conceived the idea and experimental protocol. AR and AW contributed equally to the data analysis and drafting the manuscript. YG performed the non‐parametric SCCS analysis. All authors commented and advised on all drafts of the manuscript.

## Funding Statement

During the conduct of the study, AR was funded by a population health scientist fellowship from the Medical Research Council (MR/M014649/1), ID was funded by a methodology fellowship from the Medical Research Council (G0802403/1) and LS was funded by a grant from the Wellcome Trust (098 504/Z/12/Z). The funders had no role in study design, data collection and analysis, decision to publish or preparation of the manuscript.

## Supporting information


**Appendix S1** List of *Helicobacter pylori* treatment (HPT) regimes listed in the British National Formulary
**Appendix S2** Details of propensity score model
**Appendix S3** The distribution of propensity scores by treatment group
**Appendix S4** Age bands for SCCS analysis
**Appendix S5** Assumption of the SCCS method and our approaches to dealing with them
**Appendix S6** Baseline characteristics for cohort study including those dropped from the analysis due to missing or extreme propensity score
**Appendix S7** Results of the propensity score adjusted cohort analysis using Poisson regression for all outcomes including stratification by time from exposure
**Appendix S8** Results of the self‐controlled case series analysis for first stroke
**Appendix S9** Results of the self‐controlled case series analysis for the outcomes of first MI in patients with linked HES outcome dates for multiple risk window analysis

Supporting info item
