# Supplementary material for: Evaluation of the risk of cardiovascular events with clarithromycin using both propensity score and self‐controlled study designs
Source: Br J Clin Pharmacol. 2016 Jun 8;82(2):512–21. doi: 10.1111/bcp.12983 (PMC4972168; doi:10.1111/bcp.12983)
Supplement: Supplementary file 1 — Supporting info item [file BCP-82-512-s001.docx]

Appendix S1: List of Helicobacter pylori Treatment (HPT) regimes listed in the British National Formulary

| **HPT containing clarithromycin regimes:** | **EITHER:**  clarithromycin 250mg twice daily + metronidazole 400mg twice daily  +  One of the following:  esomeprazole 20mg twice daily  lansoprazole 30mg twice daily  omeprazole 20mg twice daily  pantoprazole 40mg twice daily  rabeprazole 20mg twice daily  **OR**  clarithromycin 500mg twice daily + amoxicillin 1g twice daily  +  One of the following:  esomeprazole 20mg twice daily  lansoprazole 30mg twice daily  omeprazole 20mg twice daily  pantoprazole 40mg twice daily  rabeprazole 20mg twice daily |
| --- | --- |
| **Clarithromycin free HPT regimes:** | amoxicillin 1g twice daily + metronidazole 400mg twice daily  +  One of the following:  lansoprazole 30mg twice daily  omeprazole 20mg twice daily |

Appendix S2: Details of propensity score model

The propensity score was derived from a logistic regression model estimating the conditional probability of receiving a HPT regime containing clarithromycin given the following covariates:

| Sex |
| --- |
| Age |
| Smoking status |
| Alcohol status |
| Body Mass Index |
| Consulted GP in year before exposure |
| History of cardiovascular disease |
| History of heart failure |
| History of arrhythmia |
| History of hypertension |
| History of COPD |
| History of asthma |
| History of hyperlipidaemia |
| History of diabetes mellitus |
| History of cancer |
| History of NSAID use |
| History of oral corticosteroid use |
| History of antipsychotic use |
| History of antidepressant use |
| History of lipid lowering drug use |
| History of anticoagulant use |
| History of antiplatelet use |
| History of nitrate use |
| History of digoxin use |
| History of antiarrhythmic drug use |
| History of beta blocker use |
| History of thiazide diuretic use |
| History of calcium channel blocker use |
| History of ACEI/ARB use |
| History of loop diuretic use |

Appendix S3: The distribution of propensity scores by treatment group.

NHPT = Clarithromycin free Helicobacter pylori treatment. CHPT = Helicobacter pylori treatment containing clarithromycin. Propensity score = Propensity to be prescribed CHPT given that Helicobacter pylori treatment is prescribed.

Appendix S4: Age bands for SCCS analysis

| <20 years: | single band |
| --- | --- |
| 20-30 years: | 5 year bands |
| 30-40 years: | 2 year bands |
| 40-80 years: | 1 year bands |
| 80-90 years: | 2 year bands |
| >90 years: | single band |

Appendix S5: Assumption of the SCCS method and our approaches to dealing with them

The SCCS method relies on three key assumptions. Firstly, recurrent events must be independent—that is, having a first event does not influence the likelihood of a second event. Although this does not hold for cardiovascular events, these events are sufficiently rare that restricting the analysis to the first incident event has been shown to be a valid approach^6^.

Secondly, the occurrence of an event should not alter the probability of subsequent exposure. For example, if a recent cardiovascular event was a contraindication to prescribing HPT, it would lead to a low rate of events in the period leading up to the first prescription of HPT and may artificially exaggerate the relative rate of events occurring in exposed versus unexposed periods. This potential bias can be overcome by removing a predefined period of time before exposure from all other unexposed (baseline) time^8^. In this study a two week period before exposure was removed from the baseline period. We repeated the analysis removing a two month period before exposure from the baseline as a sensitivity analysis to ensure that the two week period was sufficient to prevent bias in our analysis.

Thirdly, the event of interest must not censor the observation period—for example, if the event increases the likelihood of death—although there is some evidence that the method is robust to this assumption.^7^ Whether this assumption is fulfilled can be readily checked by measuring short term mortality after the event of interest.

Appendix S6: Baseline characteristics for cohort study including those dropped from the analysis due to missing or extreme propensity score

| **Characteristic** | **Clarithromycin containing HPT regime** | | **Clarithromycin-free HPT regime** | |
| --- | --- | --- | --- | --- |
|  | **N (%)** | | **N (%)** | |
|  |  |  |  |  |
| Sex |  |  |  |  |
| Male | 15809 | (47.6%) | 1536 | (48.3%) |
| Female | 17429 | (52.4%) | 1646 | (51.7%) |
| Age |  |  |  |  |
| 0-40y | 7967 | (24%) | 745 | (23.4%) |
| 40-50y | 6812 | (20.5%) | 632 | (19.9%) |
| 50-60y | 6778 | (20.4%) | 681 | (21.4%) |
| 60-70y | 6315 | (19%) | 584 | (18.4%) |
| 70-80y | 3972 | (12%) | 400 | (12.6%) |
| >80y | 1394 | (4.2%) | 140 | (4.4%) |
| Smoking status |  |  |  |  |
| non-smoker | 13254 | (39.9%) | 1329 | (41.8%) |
| current smoker | 9578 | (28.8%) | 760 | (23.9%) |
| ex-smoker | 9938 | (29.9%) | 1060 | (33.3%) |
| unknown | 468 | (1.4%) | 33 | (1%) |
| Alcohol status |  |  |  |  |
| non-drinker | 5473 | (16.5%) | 565 | (17.8%) |
| ex-drinker | 1460 | (4.4%) | 153 | (4.8%) |
| current drinker (unknown quantity) | 144 | (0.4%) | 15 | (0.5%) |
| <2u/day | 5711 | (17.2%) | 562 | (17.7%) |
| 3-6u/day | 13723 | (41.3%) | 1299 | (40.8%) |
| >6u/day | 3424 | (10.3%) | 272 | (8.5%) |
| unknown | 3303 | (9.9%) | 316 | (9.9%) |
| Body Mass Index |  |  |  |  |
| normal | 12282 | (37%) | 1174 | (36.9%) |
| overweight | 11287 | (34%) | 1078 | (33.9%) |
| obese I | 4630 | (13.9%) | 484 | (15.2%) |
| obese II | 1424 | (4.3%) | 137 | (4.3%) |
| obese III | 584 | (1.8%) | 53 | (1.7%) |
| unknown | 3031 | (9.1%) | 256 | (8%) |
| Consulted GP in year before exposure | 33137 | (99.7%) | 3171 | (99.7%) |
| History of cardiovascular disease | 5356 | (16.1%) | 460 | (14.5%) |
| History of heart failure | 926 | (2.8%) | 88 | (2.8%) |
| History of arrhythmia | 1774 | (5.3%) | 164 | (5.2%) |
| History of hypertension | 10396 | (31.3%) | 992 | (31.2%) |
| History of COPD | 2129 | (6.4%) | 212 | (6.7%) |
| History of asthma | 4610 | (13.9%) | 454 | (16.3%) |
| History of hyperlipidaemia | 5848 | (17.6%) | 529 | (16.6%) |
| History of diabetes mellitus | 4701 | (14.1%) | 444 | (14%) |
| History of cancer | 6092 | (18.3%) | 554 | (17.4%) |
| History of NSAID use | 2538 | (7.6%) | 212 | (6.7%) |
| History of oral corticosteroid use | 483 | (1.5%) | 50 | (1.6%) |
| History of antipsychotic use | 1136 | (3.4%) | 96 | (3%) |
| History of antidepressant use | 3973 | (12%) | 376 | (11.8%) |
| History of lipid lowering drug use | 3878 | (11.7%) | 481 | (15.1%) |
| History of anticoagulant use | 302 | (0.9%) | 49 | (1.5%) |
| History of antiplatelet use | 2798 | (8.4%) | 341 | (10.7%) |
| History of nitrate use | 842 | (2.5%) | 76 | (2.4%) |
| History of digoxin use | 186 | (0.6%) | 24 | (0.8%) |
| History of antiarrhythmic drug use | 75 | (0.2%) | 8 | (0.3%) |
| History of beta blocker use | 2855 | (8.6%) | 286 | (9%) |
| History of thiazide diuretic use | 2407 | (7.2%) | 250 | (7.9%) |
| History of calcium channel blocker use | 2624 | (7.9%) | 280 | (8.8%) |
| History of ACEI/ARB use | 3700 | (11.1%) | 421 | (13.2%) |
| History of loop diuretic use | 915 | (2.8%) | 91 | (2.9%) |
|  |  |  |  |  |
| Total | 33238 |  | 3182 |  |

Appendix S7: Results of the propensity score adjusted cohort analysis using Poisson regression for all outcomes including stratification by time from exposure.

|  | **Patients**  **(N)** | **Patient-years** | **Events**  **(N)** | **Crude IRR**  **(95% CI)** | | | **PS Adjusted IRR**  **(95% CI)** | | |
| --- | --- | --- | --- | --- | --- | --- | --- | --- | --- |
| **First MI** |  |  |  |  |  |  |  |  |  |
| HPT containing clarithromycin | 26029 | 62118.98 | 174 | 0.89 | (0.54-1.44) | p=0.62 | 0.75 | (0.45-1.24) | p=0.26 |
| Clarithromycin free HPT | 2523 | 5688.98 | 18 | 1.00 |  |  |  |  |  |
| *Stratified Analysis by time since exposure* |  |  |  |  |  |  |  |  |  |
| *0-90 days* |  |  |  |  |  |  |  |  |  |
| HPT containing clarithromycin | 26029 | 6251.51 | 23 | 1.11 | (0.26-4.69) | p=0.10# | 0.93 | (0.22-3.98) | p=0.09# |
| Clarithromycin free HPT | 2523 | 601.60 | * | 1.00 |  |  | 1.00 |  |  |
| *91-365 days* |  |  |  |  |  |  |  |  |  |
| HPT containing clarithromycin | 24741 | 17554.55 | 39 | 0.92 | (0.33-2.56) |  | 0.77 | (0.27-2.17) |  |
| Clarithromycin free HPT | 2369 | 1649.42 | * | 1.00 |  |  | 1.00 |  |  |
| *1-2 years* |  |  |  |  |  |  |  |  |  |
| HPT containing clarithromycin | 22018 | 20519.04 | 67 | 0.61 | (0.31-1.19) |  | 0.51 | (0.26-1.01) |  |
| Clarithromycin free HPT | 2047 | 1868.93 | 10 | 1.00 |  |  | 1.00 |  |  |
| *2-3 years* |  |  |  |  |  |  |  |  |  |
| HPT containing clarithromycin | 19108 | 17793.88 | 45 | 1.98 | (0.48-8.12) |  | 1.67 | (0.40-6.94) |  |
| Clarithromycin free HPT | 1705 | 1569.03 | * | 1.00 |  |  | 1.00 |  |  |
|  |  |  |  |  |  |  |  |  |  |
| **First Stroke** |  |  |  |  |  |  |  |  |  |
| HPT containing clarithromycin | 26686 | 63847.36 | 68 | 0.38 | (0.22-0.66) | p=0.001 | 0.47 | (0.26-0.84) | p=0.01 |
| Clarithromycin free HPT | 2540 | 5746.98 | 16 | 1.00 |  |  |  |  |  |
| *Stratified Analysis by time since exposure* |  |  |  |  |  |  |  |  |  |
| *0-90 days* |  |  |  |  |  |  |  |  |  |
| HPT containing clarithromycin | 26686 | 6411.09 | 6 | 0.57 | (0.68-4.70) | p=0.56# | 0.69 | (0.08-5.82) | p=0.55# |
| Clarithromycin free HPT | 2540 | 605.63 | * | 1.00 |  |  | 1.00 |  |  |
| *91-365 days* |  |  |  |  |  |  |  |  |  |
| HPT containing clarithromycin | 25380 | 18019.87 | 20 | 0.62 | (0.18-2.07) |  | 0.75 | (0.22-2.58) |  |
| Clarithromycin free HPT | 2386 | 1663.99 | * | 1.00 |  |  | 1.00 |  |  |
| *1-2 years* |  |  |  |  |  |  |  |  |  |
| HPT containing clarithromycin | 22608 | 21086.59 | 26 | 0.26 | (0.12-0.55) |  | 0.32 | (0.14-0.70) |  |
| Clarithromycin free HPT | 2067 | 1891.22 | 9 | 1.00 |  |  | 1.00 |  |  |
| *2-3 years* |  |  |  |  |  |  |  |  |  |
| HPT containing clarithromycin | 24842 | 18329.82 | 16 | 0.46 | (0.13-1.58) |  | 0.57 | (0.16-1.99) |  |
| Clarithromycin free HPT | 1725 | 1586.14 | * | 1.00 |  |  | 1.00 |  |  |
|  |  |  |  |  |  |  |  |  |  |
| **First arrhythmia** |  |  |  |  |  |  |  |  |  |
| HPT containing clarithromycin | 26586 | 63581.67 | 95 | 0.43 | (0.26-0.69) | p=0.001 | 0.37 | (0.22-0.63) | p=0.001 |
| Clarithromycin free HPT | 2527 | 5702.77 | 20 | 1.00 |  |  |  |  |  |
| *Stratified Analysis by time since exposure* |  |  |  |  |  |  |  |  |  |
| *0-90 days* |  |  |  |  |  |  |  |  |  |
| HPT containing clarithromycin | 26586 | 6368.35 | 18 | 1.70 | (0.23-12.72) | p=0.02# | 1.49 | (0.19-11.26) | p=0.03# |
| Clarithromycin free HPT | 2527 | 602.41 | * | 1.00 |  |  |  |  |  |
| *91-365 days* |  |  |  |  |  |  |  |  |  |
| HPT containing clarithromycin | 25272 | 17943.67 | 23 | 1.06 | (0.25-4.49) |  | 0.93 | (0.22-4.00) |  |
| Clarithromycin free HPT | 2373 | 1652.76 | * | 1.00 |  |  |  |  |  |
| *1-2 years* |  |  |  |  |  |  |  |  |  |
| HPT containing clarithromycin | 22513 | 21000.53 | 26 | 0.2 | (0.10-0.38) |  | 0.17 | (0.08-0.34) |  |
| Clarithromycin free HPT | 2050 | 1874.93 | 12 | 1.00 |  |  |  |  |  |
| *2-3 years* |  |  |  |  |  |  |  |  |  |
| HPT containing clarithromycin | 19589 | 18251.12 | 28 | 0.48 | (0.19-1.25) |  | 0.42 | (0.16-1.12) |  |
| Clarithromycin free HPT | 1712 | 1572.67 | 5 | 1.00 |  |  |  |  |  |
|  |  |  |  |  |  |  |  |  |  |
| **All cause mortality** |  |  |  |  |  |  |  |  |  |
| HPT containing clarithromycin | 26827 | 64235.69 | 2621 | 1.09 | (0.95-1.25) | p=0.22 | 0.96 | (0.83-1.11) | p=0.59 |
| Clarithromycin free HPT | 2582 | 5851.81 | 219 | 1.00 |  |  |  |  |  |
| *Stratified Analysis by time since exposure* |  |  |  |  |  |  |  |  |  |
| *0-90 days* |  |  |  |  |  |  |  |  |  |
| HPT containing clarithromycin | 26827 | 6445.37 | 163 | 0.87 | (0.53-1.41) | p<0.001# | 0.78 | (0.48-1.27) | p<0.001# |
| Clarithromycin free HPT | 2582 | 615.96 | 18 | 1.00 |  |  | 1.00 |  |  |
| *91-365 days* |  |  |  |  |  |  |  |  |  |
| HPT containing clarithromycin | 25518 | 18119.60 | 359 | 0.96 | (0.68-1.36) |  | 0.86 | (0.61-1.22) |  |
| Clarithromycin free HPT | 2427 | 1692.11 | 35 | 1.00 |  |  | 1.00 |  |  |
| *1-2 years* |  |  |  |  |  |  |  |  |  |
| HPT containing clarithromycin | 22743 | 21217.86 | 327 | 1.06 | (0.72-1.56) |  | 0.95 | (0.64-1.40) |  |
| Clarithromycin free HPT | 2101 | 1924.81 | 28 | 1.00 |  |  | 1.00 |  |  |
| *2-3 years* |  |  |  |  |  |  |  |  |  |
| HPT containing clarithromycin | 19792 | 18452.86 | 1772 | 1.13 | (0.95-1.34) |  | 1.01 | (0.85-1.21) |  |
| Clarithromycin free HPT | 1761 | 1618.92 | 138 | 1.00 |  |  | 1.00 |  |  |
|  |  |  |  |  |  |  |  |  |  |
| **Cardiovascular mortality** |  |  |  |  |  |  |  |  |  |
| HPT containing clarithromycin | 11616 | 27729.71 | 416 | 1.05 | (0.73-1.50) | p=0.80 | 0.93 | (0.64-1.34) | p=0.69 |
| Clarithromycin free HPT | 1058 | 2234.28 | 32 | 1.00 |  |  | 1.00 |  |  |
| *Stratified Analysis by time since exposure* |  |  |  |  |  |  |  |  |  |
| *0-90 days* |  |  |  |  |  |  |  |  |  |
| HPT containing clarithromycin | 11616 | 2787.91 | 30 | 0.67 | (0.24-1.90) | p<0.001# | 0.61 | (0.21-1.73) | p<0.001# |
| Clarithromycin free HPT | 1058 | 249.05 | * | 1.00 |  |  | 1.00 |  |  |
| *91-365 days* |  |  |  |  |  |  |  |  |  |
| HPT containing clarithromycin | 11039 | 7832.93 | 57 | 1.21 | (0.44-3.34) |  | 1.10 | (0.40-3.05) |  |
| Clarithromycin free HPT | 969 | 665.87 | * | 1.00 |  |  | 1.00 |  |  |
| *1-2 years* |  |  |  |  |  |  |  |  |  |
| HPT containing clarithromycin | 9841 | 9180.91 | 43 | 0.57 | (0.24-1.34) |  | 0.52 | (0.22-1.23) |  |
| Clarithromycin free HPT | 810 | 730.63 | 6 | 1.00 |  |  | 1.00 |  |  |
| *2-3 years* |  |  |  |  |  |  |  |  |  |
| HPT containing clarithromycin | 8553 | 7927.96 | 286 | 1.18 | (0.73-1.90) |  | 1.07 | (0.66-1.75) |  |
| Clarithromycin free HPT | 647 | 588.73 | 18 | 1.00 |  |  | 1.00 |  |  |

IRR = Incidence rate ratio, CI = confidence interval, HPT = Helicobacter pylori Treatment MI = myocardial infarction, #LRT for interaction. *where there are less than 5 patients the exact

number has been withheld in accordance with the confidentiality rules of the CPRD

Appendix S8: Results of the self-controlled case series analysis for first stroke.

|  | **Patients**  **(N)** | **Patient-years** | **Events**  **(N)** | **Age adjusted IRR**  **(95% CI)** | |  |
| --- | --- | --- | --- | --- | --- | --- |
| *Single risk window* |  |  |  |  |  |  |
| Baseline | 348 | 4560.9 | 306 | 1 |  |  |
| 1 year post-exposure | 348 | 350.48 | 40 | 1.33 | (0.93-1.89) | p=0.11 |
| *Multiple Risk window* |  |  |  |  |  |  |
| Baseline | 347 | 3945.24 | 246 | 1 |  |  |
| day 1-30 post-exposure | 348 | 30.13 | * | 1.27 | (0.4-4) | p=0.68 |
| day 31-90 post-exposure | 348 | 59.61 | 6 | 1.27 | (0.56-2.89) | p=0.57 |
| day 91-365 post exposure | 345 | 261.66 | 31 | 1.47 | (0.99-2.19) | p=0.06 |
| year 1-2 post-exposure | 326 | 321.48 | 35 | 1.47 | (1.01-2.13) | p=0.04 |
| year 2-3 post-exposure | 301 | 293.34 | 25 | 1.12 | (0.73-1.72) | p=0.6 |

All IRRs are age adjusted and derived from conditional Poisson regression. MI = myocardial infarction, CI = confidence interval, IRR = incidence rate ratio. Median follow up was 14.0 years. *where there are less than 5 patients the exact number has been withheld in accordance with the confidentiality rules of the CPRD

Appendix S9: Results of the self-controlled case series analysis for the outcomes of first MI in patients with linked HES outcome dates for multiple risk window analysis.

|  | **Patients**  **(N)** | **Patient-years** | **Events**  **(N)** | **Age adjusted IRR**  **(95% CI)** | |  |
| --- | --- | --- | --- | --- | --- | --- |
| **Primary Outcome: First MI**  **(median follow up 13.6y)** |  |  |  |  |  |  |
| Baseline | 359 | 4111.38 | 250 | 1 |  |  |
| day 1-30 post-exposure | 359 | 30.19 | 8 | 3.77 | (1.85-7.68) | p<0.001 |
| day 31-90 post-exposure | 357 | 59.21 | 7 | 1.67 | (0.78-3.58) | p=0.18 |
| day 91-365 post exposure | 352 | 260.83 | 21 | 1.13 | (0.72-1.79) | p=0.59 |
| year 1-2 post-exposure | 335 | 318.93 | 32 | 1.40 | (0.99-2.06) | p=0.08 |
| year 2-3 post-exposure | 305 | 296.07 | 31 | 1.49 | (1.01-2.20) | p=0.04 |
|  |  |  |  |  |  |  |

All IRRs are age adjusted and derived from conditional Poisson regression. MI = myocardial infarction, CI = confidence interval, IRR = incidence rate ratio
